# In Search of the Factors Behind Naive Sentence Judgments: A State Trace Analysis of Grammaticality and Acceptability Ratings

**DOI:** 10.3389/fpsyg.2019.02886

**Published:** 2019-12-20

**Authors:** Steven Langsford, Rachel G. Stephens, John C. Dunn, Richard L. Lewis

**Affiliations:** ^1^Department of Psychology, University of Michigan, Ann Arbor, MI, United States; ^2^Department of Psychology, University of Adelaide, Adelaide, SA, Australia; ^3^Psychological Science, University of Western Australia, Perth, WA, Australia

**Keywords:** acceptability, grammaticality, state trace analysis, rating task, language modeling

## Abstract

We present a state-trace analysis of sentence ratings elicited by asking participants to evaluate the overall acceptability of a sentence and those elicited by asking participants to focus on structural well-formedness only. Appealing to literature on “grammatical illusion” sentences, we anticipated that a simple instruction manipulation might prompt people to apply qualitatively different kinds of judgment in the two conditions. Although differences consistent with the subjective experience of grammatical illusion dissociations were observed, the state trace analysis of the rating data indicates that responses were still consistent with both judgment types accessing a single underlying factor. These results add to the existing comparisons between analytic and probabilistic modeling approaches to predicting rating judgments.

## Introduction

Language communities have been shown to be consistent and reliable in their consensus reporting of how acceptable a sentence is (Sprouse et al., 2013; Mahowald et al., 2016). Quantifying, predicting, and contrasting ratings based on such judgments has for a long time been an important part of linguistics research (Schütze and Sprouse, 2014). But despite high agreement about *what is acceptable*, it is not at all obvious *what acceptability is*. Plausible candidates include the processing effort required (Braze, 2002; Hofmeister et al., 2013), the probability of the sentence under some appropriate language model (Chater and Manning, 2006), an expanded notion of probability including the naturalness/oddity given situational pragmatics (Masia, 2017; Domaneschi and Di Paola, 2018), or a combination of error signals that arise from different component stages of language processing (Sprouse, 2018).

Probably the most popular view is that acceptability is a combination of error signals from all these sources, which could of course include processing effort and word co-occurrence statistics as particularly salient signals (Sprouse, 2018). From this general perspective, a full understanding of acceptability ratings would entail describing the factor structure of linguistic acceptability and specifying how the different components interact.

### Why Care About the Factor Structure of Ratings?

An understanding of the factor structure underlying sentence ratings may be helpful in interpreting conflicts between crowd-sourced acceptability judgments and sentence-status descriptions arrived at by analysis or other means. One such situation arises when crowd-sourced acceptability judgments conflict with descriptions of grammatical status arrived at by analysis or other means (Sprouse et al., 2013). How should these results be interpreted? Assuming that best practices have been followed to protect the reliability of the rating data (Myers, 2009; Ahler et al., 2019) one possible interpretation is that the analysis is in error. However, this is not the only interpretation. It is possible that the analysis and the acceptability judgment simply reflect distinct properties of the sentence, with acceptability responsive to a range of additional factors outside the scope of the analysis. Using structured interviews, Schütze (in press) finds that this is the case for at least some items identified by Sprouse et al. (2013) as examples of inconsistency between expert analysis and crowd-ratings. Schütze (in press) calls for qualitative data about the motivation for a rating to be collected alongside likert-style judgments, in order to identify the interpretation a rating was made under and any special features influencing the rating, such as an unknown word. It is possible that detailed instructions about the target property to be rated could reduce the variation in rating motivation. The study described below contrasts different instructions, giving an example of the size of such instruction-based effects.

Another arena in which the factor structure of judgment data is important is when it is used in the design and evaluation of language models. This usage could be direct, in a supervised learning system predicting acceptability ratings on hold-out items from a collection of rated sentences (Warstadt et al., 2019), or indirect, when the ability to predict sentence acceptability judgments is used to evaluate an unsupervised learning system trained on unannotated corpora (Lau et al., 2017). In either case, the composition of factors underlying ratings are important to the interpretation of the results. If sentence ratings are responsive to multiple properties of a sentence, for example both “surface probability” and “structural soundness,” it is possible that evaluating models on their ability to predict ratings will lead to models that privilege one component at the expense of the other. A concrete example of this kind of feature-substitution appears in the computer vision literature, where convolutional neural nets have been found to weight texture more heavily than shape (Geirhos et al., 2018). This feature weighting is the exact opposite of the human pattern, but it arises naturally in this context because texture is highly predictive of object identity in the training data and involves short-range dependencies that are easier for these learning architectures to discover. To the extent that modern language modeling relies on similar learning architectures, it is similarly vulnerable to under-weighting or even omitting the “shape-like” properties of natural language if “texture-like” properties are available in rating judgments (Warstadt and Bowman, 2019).

One potential example of this scenario in linguistics is presented by Sprouse et al. (2018) in response to work by Lau et al. (2017) (see also Lappin and Lau, 2018). In brief, Sprouse et al. (2018) distinguishes between three different performance metrics in order to compare models presented by Lau et al. (2017) with existing theories of syntax as represented by submissions to *Linguistic Inquiry* and Adger's *Core Syntax* (Adger, 2003). One metric, the gradient metric, is a correlation between predicted rating and observed rating. Another, the categorical metric, is a discretized version of the gradient metric based only on the rank order of items. A third, the experimental-logic metric, counts successful predictions for the presence or absence of a difference in rating between two carefully controlled comparison items. The three performance measures are related: given a scheme for predicting rating scores for any sentence, the categorical and experimental-logic metrics are discretized versions of differences under the gradient metric. Despite this close relationship, Sprouse et al. (2018) report that high performance under the gradient metric is not necessarily associated with similarly high performance under the categorical and experimental-logic metrics. A striking feature of this work is the demonstration that categorical distinctions derived from the linguistic literature perform well on the two discrete metrics for which they are applicable but are not able to give predictions on the gradient metric, while a probabilistic model with attested high performance on the gradient metric shows a drop in performance when evaluated on the categorical and experimental logic metrics. One possible interpretation of this dissociation in performance might be that different linguistic properties are accessed by corpus-trained probabilistic models and expert analysis.

A second motivation for the study presented below is to explore contrasting explanations for what Sprouse et al. (2018) describe as a trade-off between performance on the gradient metric and the categorical metric. The suggestion that this performance trade-off reflects attention to different linguistic properties seems well-motivated on theoretical grounds, but in principle such dissociations can appear even if there is only one key well-formedness factor underlying both metrics (Loftus, 1978). In particular, we note that Bader and Häussler (2010) have explored a principled mapping between gradient and categorical judgments of grammatical status directly relevant to the distinction Sprouse et al. (2018) draws between the categorical and gradient evaluation metrics. The mapping scheme is an implementation of signal detection theory, and as such draws on a well-established tradition of such models in psychophysics (Green and Swets, 1966). This class of models contains a mechanism whereby responses can produce apparent dissociations even when both are based on the same latent factor (Stephens et al., 2018, 2019). Such an account would still be consistent with the performance contrasts demonstrated by Sprouse et al. (2018). Under a signal-detection account, a change in response thresholds, a change in noise levels, or both in concert could in principle produce differences like those observed between the discrete and gradient evaluation metrics even if both reflect a single underlying well-formedness factor. Proposing a single-factor account of differences between expert analyses and probabilistic models may seem extreme given the extensive theoretical differences between these approaches. We raise the possibility to emphasize the way uncertainty about the factor structure of acceptability rating judgments leaves unclear what kind of extension to the modeling work of Lau et al. (2017) would be the most natural response to the variable pattern of performance across evaluation metrics and probe sentences described by Sprouse et al. (2018).

### A Manipulation Targeting Latent Factors

In this study we use a simple instruction manipulation to contrast the ratings produced in response to two different questions. One question type asked participants to rate the acceptability of the target sentence, and one asked them to indicate how confident they were that the sentence was grammatical. We ask whether a representative sample of American English speakers would make any distinction at all between these two questions, and if so, what changes in the decision making process might underlie the distinction. The hypothesis that qualitatively different types of judgment might be elicited is suggested by the grammatical illusion literature, to the extent that the striking dissociation between syntactic soundness and acceptability evident in grammatical illusions is thought to be apparent to audiences without extensive training in linguistics. Alternatively, people may not distinguish between the two questions at all, or they may respond with a distinction that has no special relationship with syntactic soundness, such as a uniform reduction in ratings for all sentences in one condition, a move toward more extreme ratings for all sentences, or a change in noise levels. The main goal of this study is to differentiate between these possible scenarios.

To make the contrast between the two question types as salient as possible, we chose a within-subjects design, with each participant giving two blocks of ratings, one for each instruction condition. Items were never rated twice by any one participant. Participants were introduced to the idea of isolating structure from other components of overall acceptability with a brief description of the “colorless green ideas sleep furiously” sentence (Chomsky, 1957)^1^ and then asked to rate one block of sentences for overall acceptability and one block for grammatical validity only.

In order to expose the relationship, if any, between the lay interpretation of the two different questions and the distinction drawn between acceptability and structural soundness in linguistics, we presented sentence types commonly described as particularly strong examples of the theoretical dissociation.

One particularly well-known example is center-embedding, which produces sentences widely regarded as grammatical but unacceptable (Chomsky and Miller, 1963; Karlsson, 2007). There are also ungrammatical sentences with unusually high acceptability. Possibly the most well-known is the comparison illusion (Phillips et al., 2011), often illustrated with the example “More people have been to Russia than I have.” This sentence is considered unparsable because it has no possible interpretation, and cannot be considered either true or false in any possible state of the world. However, it is generally considered to be more acceptable than might be expected of a nonsense sentence and given the status of a “grammatical illusion.” Other phenomena thought to introduce acceptability differences between sentences with equivalent grammatical status include negative polarity item (NPI) illusions (Drenhaus et al., 2005) and agreement attraction sentences (Bock and Miller, 1991). In addition to stimuli constructed to replicate these phenomena, we also examined a set of stimuli drawn from those used in Sprouse et al. (2013) for which expert judgment apparently differed from crowd-sourced judgments, hypothesizing that the difference may have been because different judgment types were applied. The full set of stimuli used are given in Appendix B.

### State Trace Analysis

To interpret the impact of the instruction manipulation we turn to state-trace analysis (Bamber, 1979; Kalish et al., 2016). State-trace analysis is a tool for identifying dissociable sub-systems in task performance. The “state-trace” at the heart of this analysis is a plot of the co-variation of two dependent variables across different experimental conditions (see Newell and Dunn, 2008 for a review, Dunn and Kalish, 2018 for a more complete treatment). Mathematically, a state trace is a generalization of the yes-no receiver-operating-characteristic curve, a standard tool for evaluating classification accuracy that describes the full range of possible trade-offs between sensitivity and specificity (Bamber, 1979). Under relatively weak assumptions, a state trace plot can be diagnostic of the dimensionality of the underlying process. Single process or single resource accounts, by definition, claim that all possible pairs of outcomes can be described as a point on a single underlying dimension. In this case, points on the state-trace plot are restricted to fall on a one-dimensional line. In contrast, if there are multiple processes or mental resources underlying task performance, points on the state trace plot are not so constrained, and are overwhelmingly more likely to “break the line” than not. Various frequentist (Kalish et al., 2016) and Bayesian (Prince et al., 2012; Davis-Stober et al., 2016; Cox and Kalish, 2019) formulations for state trace analyses exist, but in essence all report on whether or not it is possible to conclude that the “line has been broken” while allowing for noisy measurement. The implementation used here is the frequentist one due to Kalish et al. (2016). This test takes the one-dimensional scenario as the null hypothesis and produces a *p*-value representing how extreme the observed data are in a bootstrapped population of simulated outcomes drawn from the null. More detail regarding the bootstrap procedure underlying the *p*-value reported can be found in Wagenmakers et al. (2004). The state trace analysis described in Kalish et al. (2016) uses the data-informed variant of this procedure. A sampling distribution over the difference in fit for the one-dimensional and two-dimensional models is generated using bootstrapped samples drawn from the full data set. At each iteration, goodness of fit values are calculated using a coupled monotonic regression as described by Burdakov et al. (2012). The *p*-value is the proportion of goodness-of-fit differences observed with bootstrapped samples that exceed the difference observed in the full sample. Following the normal logic of *p*-values, if this proportion is large, the observed result is unremarkable under the null hypothesis, while if it is small the observed data constitute an extreme observation if the one-dimensional account were true. Full implementation details appear in Kalish et al. (2016). The motivation and foundations of the procedure are discussed further in Dunn and Kalish (2018). An accessible discussion of applications in psychology appears in Newell and Dunn (2008).

Although in principle any set of data points can be fit by some sufficiently complex one-dimensional line, because the experimental conditions are under the researcher's control they can be selected in order to produce a monotonic relationship with the outcome variables. This assumption of monotonicity has to be defended on its own merits for each application (Ashby, 2014), but if it can be assumed it imposes a severe constraint on the state trace plot. Under these conditions, single process accounts commit to predicting a monotonic state trace. Multiple process accounts can in principle also produce monotonic state traces, but their extra degrees of freedom allow for so many alternative possibilities that the one dimensional result is relatively unlikely and constitutes an extreme observation (for a frequentist) or a highly suspicious coincidence (to a Bayesian).

**Figure 1:**
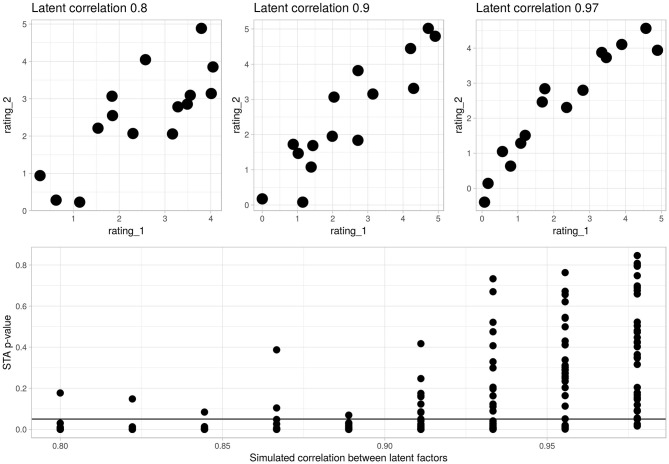
Illustrative state-trace analyses on simulation data. The lower panel presents *p*-values from 315 simulated experiments where ratings type 1 and 2 were correlated to the degree specified on the x-axis. The horizontal bar is at *p* = 0.05. There are 35 repetitions at each degree of correlation. The upper panel presents three representative state-trace plots from simulations run at 0.8, 0.9, and 0.977. The state trace analysis tends to reject the null hypothesis of a single latent factor when the simulated factors are correlated at <0.9, and often fails to reject above that level. It is important to note that the state trace analysis is not just a test of correlation: by considering the consistency of item ranks it avoids assuming linearity.

To illustrate the application of state trace analysis, Figure 1 presents the results of a series of analyses on simulation data. These simulations were generated by taking the actual experimental data (described below) and substituting simulated responses for the observed ones. In these simulations, each sentence type had associated with it two latent properties specifying the true consensus mean for type-1 ratings and a type-2 ratings. The property underlying type-1 ratings was drawn from a uniform distribution between 0 and 5 (the range of the rating scale used in the study), the property underlying type-2 ratings added gaussian noise to produce a random variable linked to the type-1 property by a specified level of correlation. Observed ratings of each type were generated by adding gaussian noise with standard deviation 1 to the true consensus rating for each sentence. As the correlation between the two latent properties approaches 1, the simulation behaves more and more like a one-dimensional process, the state trace plot produces a thinner one-dimensional line, and the *p*-values associated with the state trace analysis approach a uniform distribution. Conversely, as the correlation between the two latent properties decreases the state trace plot produces a fatter two-dimensional ribbon shape, and the *p*-values associated with the state-trace analysis tend to be confined to low values. A critical advantage of using a state trace analysis over simply examining the correlation between the two rating types is that the state trace analysis is not constrained by an assumption of linearity. By considering only the rank order of items under each rating, it tests for the monotonicity rather than the linearity of a relationship, and makes no distributional assumptions. Since the simulation pictured in Figure 1 does not vary the form of the relationship between simulated rating type-1 and rating type-2, the distribution of simulated *p*-values in this plot may not reflect the true power of the experimental design. We present it here as a reference for readers who may be unfamiliar with state trace plots.

State trace analyses have been applied effectively across a number of different domains in cognitive science, where questions about the number of processes underlying a phenomenon are common. Example applications include in memory (Dunn, 2008), face recognition (Prince and Heathcote, 2009), and reasoning (Stephens et al., 2018).

### Summary

This study presents a state trace analysis of judgment data collected under an instruction manipulation contrasting judgments about sentence structure specifically with judgments about overall sentence acceptability. We use a within-subjects design, and collected probe sentences thought to maximize the distinction between structural and other contributors to overall rating judgments. We consider detailed instructions as complementary to the qualitative review of ratings advocated by Schütze (in press), and the results of this study give a sense of the order of magnitude of instruction-driven effects. By applying a state-trace analysis, we are also able to comment on the interpretation of the different evaluation metrics described by Sprouse et al. (2018) and their implications for language modeling. To foreshadow the results, we do observe differences in ratings for at least some probe sentences that align with the way the linguistics literature typically separates structural features from overall acceptability. Specifically, we find that errors of agreement attraction are rated more leniently for acceptability than grammatical soundness, and center-embedding sentences are rated somewhat more leniently for grammatical soundness than acceptability (although overall ratings for this sentence type are consistently low). Since these differences are in opposite directions, they cannot be accounted for by a simple scaling relation. However, a state-trace analysis of the relationship between the two rating types suggests that they are plausibly monotonically related, leaving open the possibility that a single well-formedness factor underlies both kinds of ratings.

## Method

The experiment presented instructions asking each participant to rate sentences in two distinct question blocks, one asking about acceptability and the other grammatical soundness. The order of question types was randomized. Each block contained 30 test items. The grammatical soundness block contained two additional attention check items which were excluded from analysis. We turned to the literature on grammatical illusions (Phillips et al., 2011) to find sentence types known to produce striking contrasts between their acceptability and grammatical status. We examined doubly center-embedded relative clauses (Chomsky and Miller, 1963), NPI illusions (Drenhaus et al., 2005), agreement attraction sentences (Bock and Miller, 1991), and comparative illusions (Townsend and Bever, 2001). In addition, we also examined a set of stimuli drawn from those used in Sprouse et al. (2013) for which expert judgment apparently differed from crowd-sourced judgments, hypothesizing that the difference may have been because different judgment types were applied. The full set of stimuli used are given in Appendix B.

### Stimuli

There were 433 sentences included in the stimuli pool. Of these, 112 were center-embedding sentences, 48 based on stimuli used in Gibson and Thomas (1999), and 64 from Vasishth et al. (2010). Each center-embedding sentence had four variations, one grammatical full version and three ungrammatical partial versions derived by deleting either the first, second, or third verb phrase. An example is “The ancient manuscript that the grad student who the new card catalog had confused a great deal was studying in the library was missing a page.” from which “had confused,” “was studying,” or “was missing a page” can be deleted to create a set of four related sentences. We anticipated that the grammatical full center-embedding would be considered relatively low on acceptability. This sentence structure also shows acceptability differences among the ungrammatical variations. In English, deleting the second verb phrase can improve ratings for this sentence type (Vasishth et al., 2010).

There were 124 agreement attraction sentences, all based on prompts used in Bock and Miller (1991). Each agreement attraction sentence had four variations, grammatical singular-singular agreement, ungrammatical singular-plural clashes, ungrammatical plural-singular clashes, and grammatical plural-plural agreement. An example is “The slogan on the poster is offensive to vegetarians,” which with the variations slogan/posters, slogans/poster, and slogans/posters creates a set of four sentences. Although errors are relatively rare in natural language use, agreement attraction errors are among the more common types appearing in English (Bock, 2011) and were anticipated to receive high acceptability ratings alongside low grammaticality ratings.

There were 69 NPI sentences. These were original stimuli intended to follow the NPI illusory licensing pattern (Drenhaus et al., 2005) with reference to example sentences described in Xiang et al. (2009). Each NPI sentence was given in grammatical “valid licensing” and ungrammatical “partial match” and “unlicensed” forms. One example is “No restaurants that local newspapers have recommended in their dining reviews have ever gone out of business” (valid). “The restaurants that no local newspapers have recommended in their dining reviews have ever gone out of business” (partial match). “Most restaurants the local newspapers have recommended in their dining reviews have ever gone out of business” (unlicensed). The partial match and unlicensed forms were anticipated to give different acceptability ratings despite similar (poor) grammaticality status.

There were 48 comparison illusion sentences, drawn from those used by Wellwood et al. (2018). Each sentence had two variations, one grammatical with compatible comparisons and one ungrammatical illusion sentence with incompatible comparisons. One example is the pair of sentences “Last summer more famous bands had a big stadium show than lesser-known bands did.” and “Last summer more famous bands had a big stadium show than the lesser-known band did.” Although the form with incompatible comparisons is ungrammatical and admits no possible interpretation, these sentences are often considered to have strikingly high acceptability.

Finally, there were 80 sentences drawn from stimuli used in Sprouse et al. (2013). This study compared the status assigned to sentences by contributors to the journal Linguistic Inquiry (conforming or non-conforming to a particular linguistic pattern under discussion) with acceptability ratings given by naive participants. Although strong agreement was the rule across the majority of items, the sentences used here were drawn from the minority of items for which there was disagreement, i.e., non-conforming items that received above-median acceptability ratings (20 sentences) or conforming items that received below-median acceptability ratings (60 sentences). Unlike the other sentence types, these sentences were heterogeneous in structure. One example of a poorly-rated but pattern-conforming sentence is “We proved Susan to the authorities to be the thief.” One non-conforming but highly-rated sentence is “All the postal workers seem to have all taken a break at the same time.” Unlike the other stimuli considered here, none of these sentences have been claimed to produce “illusions” directly dissociating the acceptability and grammatical status of any single item. However, we considered it possible that the apparent conflict between the pattern conforming/violating status of these examples and their crowd-sourced acceptability scores is that the two reflect different kinds of judgment.

### Presentation

Stimuli were presented to participants as a web page. The landing page contained a consent preamble, after which participants were given instructions describing the two kinds of judgments they would be asked to make. The instructions are given in full in Appendix A. Grammaticality judgments were described as rating the participant's confidence in whether or not an item “follows the rules” for constructing an English sentence. Participants were asked to “label all sentences with a grammatical error as ungrammatical, even if the error is small, and label all sentences with no errors as grammatical, even if they are badly written or unclear.” Acceptability was described as a broader concept “more about how natural a sentence sounds.” with the explanation that “Among all grammatical sentences, some will be highly acceptable and ‘sound good’ while others will be not very acceptable and ‘sound bad,’ even though they're all grammatical. Similarly, although ungrammatical sentences tend to ‘sound bad,’ some are worse than others.” Participants needed to pass a comprehension quiz to progress from the instructions to the study task. This consisted of a two-item multiple-choice quiz that asked “For this study, which of these best describes a **grammatical** sentence?” with the expected answer “A sentence that ‘follows the rules’ of English, whether it makes sense or not.” and “For this study, which of these best describes an **acceptable** sentence?” with the expected answer “A sentence that ‘sounds good,’ or ‘sounds natural’.” Multiple attempts were allowed, however failed attempts returned participants to the beginning of the instruction sequence. Before continuing to the study task, participants were asked to self-report age, gender, native language, and current country of residence. Each question block was preceded by a short prompt screen recalling the instructions. For the grammaticality judgement block, the prompt screen read “This block of questions asks you to judge if a sentence is grammatical or not. It doesn't matter if the sentence is ugly or even makes no sense: please answer ‘Yes’ if it is a valid construction in English or ‘No’ if it is not.” For the acceptability judgment block, the prompt screen read “This block of questions asks you to judge how acceptable a sentence is. Here ‘acceptable’ means ‘well-formed’ or ‘natural sounding.’ The sentences here range in acceptability from very good to very poor, please use the rating scale to indicate where each sentence falls in this range.” The order of blocks was randomized. Trials displayed a html h1 title with the current question type, either “Is this a valid grammatical English sentence?” or “Is this an acceptable English sentence?” Centered under the title was a box with a 1px solid green border containing the test sentence. The dimensions of this box may have varied depending on participant's device and browser, font size was 1.5 em. Response options were displayed under the test item outside this bounding box, and consisted of an evenly spaced row of six html buttons. In the grammaticality judgment block these were labeled “Definitely not grammatical,” “Probably not grammatical,” “Possibly not grammatical,” “Possibly grammatical,” “Probably grammatical,” and “Definitely grammatical.” In the acceptability judgment block they were labeled “Highly unacceptable,” “Unacceptable,” “Somewhat unacceptable,” “Somewhat acceptable,” “Acceptable,” and “Highly acceptable.” This labeling for the response options does introduce a difference between instruction conditions, in that the “grammaticality” judgment is presented as a rating of confidence while the “acceptability” judgment is presented as one of degree. This design choice allowed us to describe “grammaticality” to participants as a categorical structural property without varying the number of responses available.

Participants progressed to the next item immediately on making each response. The response buttons were disabled for the first 1,000 ms of each trial. Each of the two blocks consisted of 30 probe items randomly drawn from the pool of stimuli, with the “grammatical” judgement block also containing two additional attention check items. Item draws were without replacement so participants never viewed the same sentence twice. Randomization was uniform over sentence type, the main unit of analysis, rather than uniform over items. The attention check items added to the “grammatical” judgment block were identical for every participant: “Sarah expected to get a good grade.” and “Him would have been fired.” These were considered to have known grammatical status (high and low, respectively) and were used as attention checks, triggering the exclusion of participants who gave an unexpected rating to either item. These attention check items were not included in the main analysis.

### Participants

324 participants were recruited via Amazon Mechanical Turk. Ages ranged from 18 to 71, with a mean age of 35, 126 female and 4 declining to give a gender. We interpret these responses as indicating our sample is an typical of the Mechanical Turk workplace, but note that as well as being extremely WEIRD in the sense of Henrich et al. (2010), the MTurk worker pool has a slightly higher average education than the US population at large (Levay et al., 2016), which may be relevant to the interpretation of the instruction manipulation. A relatively large proportion of recruited participants were excluded from analysis. Five reported being non-native English speakers, 26 did not complete all questions, 61 completed with unrealistically fast response times (defined as <4 min), and 108 gave unexpected answers to the attention check questions, either failing to use one of the lowest two response options for “Him would have been fired” or failing to use one of the highest two response options for “Sarah expected to get a good grade.” Fifty-five participants triggered multiple exclusion criteria, in total 135 recruited participants were excluded and 189 retained (58% retention). Mean total participation time was ~10 min, including time spent reading the instructions. Mean response time per-item was 7.7 s.

### Results

On average each sentence received 13 ratings in each instruction condition, the first and third quartiles were 8 and 18 ratings per item, respectively. Ratings were coded between 0 and 5.

#### Response to Instructions

A natural first question is whether the instruction manipulation produced any difference in responding at all.

**Figure 2:**
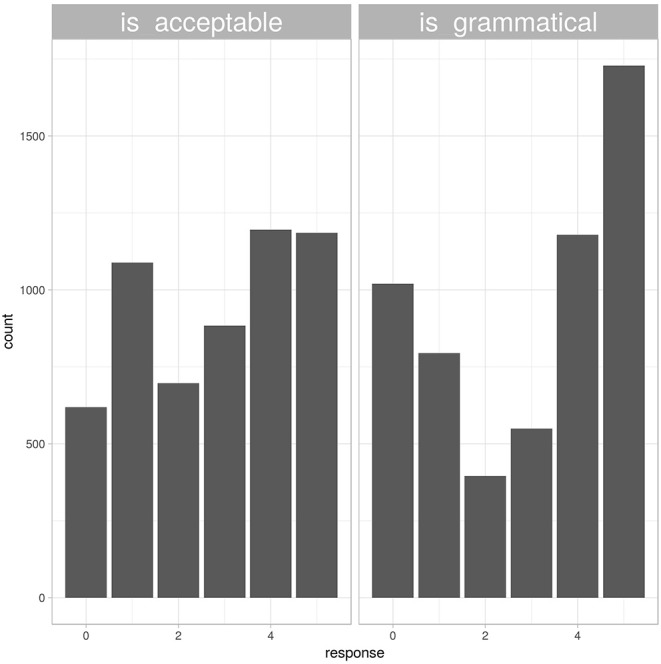
Histogram of responses from different instruction blocks. Responses to the question “is this an acceptable sentence” (left panel) were more uniformly distributed than responses to the question “is this a grammatical sentence” (right panel).

Responses to the grammaticality question were both slower and more extreme than responses to the acceptability question. After standardizing response times for each participant, responses to grammaticality questions were on average 0.1 standard deviations slower than participants' overall mean response time, while responses to acceptability questions were 0.01 standard deviations faster. This difference was statistically significant (*t*_*df* = 11231_ = −6.62, *p* < 0.001). Responses to the grammaticality question were also numerically more extreme, as shown in Figure 2. Only 31% of responses to the “acceptability” question used the most extreme options in either direction, compared to 48% of responses to the “grammatical” question.

**Figure 3:**
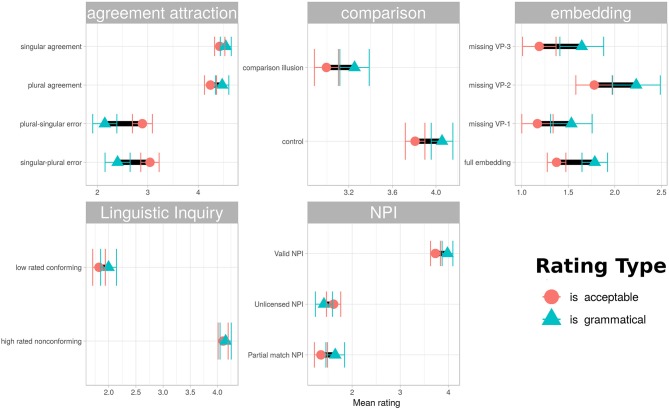
Mean ratings for various sentence types under the two instruction conditions. Intervals are 95% confidence. Although ratings under the two different instruction types were strongly correlated, the instructions did appear to produce relative differences, with some sentences receive more lenient ratings under one or the other instruction condition. Agreement attraction errors were rated more leniently under “acceptability” instructions than “grammaticality” instructions, while center-embedding sentences showed the reverse pattern, being more leniently rated under “grammaticality” instructions. Comparison sentences were more leniently rated under “grammaticality” instructions, but the difference between the two rating types was similar for both illusion and control sentences. The NPI items and those drawn from Linguistic Inquiry articles received similar ratings under both instructions.

The sentence types used in this stimulus set were chosen to give the best possible chance of dissociating ratings emphasizing structural well-formedness from those based on overall acceptability as described in the instructions. Agreement attraction sentences were hypothesized to be highly acceptable, even in their ungrammatical variations. Center-embedding sentences were predicted to be rated as grammatical but unacceptable, and the missing VP2 variation was expected to receive more favorable acceptability ratings while having the same grammatical status as the other missing verb-phrase variants. Comparison illusion sentences were expected to be higher in acceptability ratings than grammaticality ratings for the incompatible-comparison variation only. NPI illusion sentences were expected to be rated as more acceptable under partial match than unlicensed variations, with both having low grammaticality ratings. The pattern-conforming and non-conforming examples from Linguistic Inquiry articles were expected to receive ratings more in line with their pattern-conforming status when rated under grammaticality instructions than acceptability instructions. Figure 3 summarizes the differences found graphically, with the corresponding mean ratings and *t*-tests for the difference between means given in Table 1.

**Table 1:** Means and significance tests corresponding to Figure 3.

**Phenomenon**	**Item type**	**Mean acceptability**	**Mean grammaticality**	***t*-test**
Linguistic Inquiry	Low rated conforming	1.8	2	No difference
Linguistic Inquiry	High rated non-conforming	4.1	4.2	No difference
Embedding	Full embedding	1.4	1.8	*t*_*df* = 1000_ = −4.7,p = 2.6e−06
Embedding	Missing VP-2	1.8	2.2	*t*_*df* = 360_ = −2.7, *p* = 0.0069
Embedding	Missing VP-3	1.2	1.6	*t*_*df* = 350_ = −3, *p* = 0.0027
Embedding	Missing VP-1	1.2	1.5	*t*_*df* = 380_ = −2.6, *p* = 0.011
Agreement attraction	Plural-singular error	2.9	2.1	*t*_*df* = 550_ = 4.7,p = 3.2e−06
Agreement attraction	Plural agreement	4.2	4.5	*t*_*df* = 550_ = −2.6, *p* = 0.01
Agreement attraction	Singular agreement	4.4	4.5	No difference
Agreement attraction	Singular-plural error	3	2.4	*t*_*df* = 520_ = 4.1,p = 5e−05
NPI	Valid NPI	3.7	4	*t*_*df* = 1100_ = −3.2, *p* = 0.0013
NPI	Partial match NPI	1.4	1.6	*t*_*df* = 490_ = −2.5, *p* = 0.014
NPI	Unlicensed NPI	1.6	1.4	No difference
Comparison	Control	3.8	4.1	*t*_*df* = 1100_ = −3.6, *p* = 0.00032
Comparison	Comparison illusion	3	3.3	*t*_*df* = 1100_ = −2.9, *p* = 0.0038

*Most differences between rating types are significant at p < 0.01 level. One exception was the items drawn from examples used in Linguistic Inquiry articles, which received indistinguishable ratings under both instructions*.

**Figure 4:**
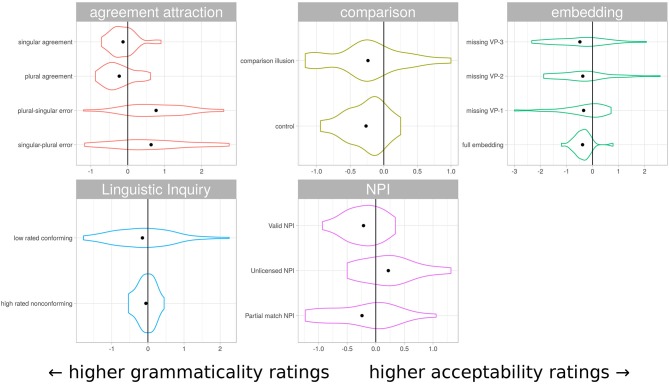
Distribution of rating differences under different instruction sets. Each violin represents the distribution of mean item rating difference under the different instruction conditions. All differences are *acceptabilityrating* − *grammaticalityrating*, so items rated more favorably under grammaticality instructions tend to the left. This visualization represents the same data as Figure 3, but obscures absolute rating magnitude and displays richer information about the distribution of differences. The sentence classes canonically regarded as grammatical typically have much lower variability in difference scores.

It is clear that the characterization of sentences as acceptable-but-not-grammatical or grammatical-but-not-acceptable is not reflected in an absolute sense in these ratings. However, in at least some cases participants appeared to distinguish between the instruction sets selectively for particular sentence types. Agreement attraction sentences were rated more leniently under acceptability instructions than grammaticality instructions, while center-embedding sentences showed the reverse pattern. Figure 4 shows an alternative visualization of the differences, plotting the distribution of rating differences for individual items under different instruction conditions, grouped by sentence structure type. This view emphasizes the difference in variability masked by the more conventional comparison of mean ratings in Figure 3. Sentences canonically regarded as grammatical show markedly less variability than their ungrammatical counterparts.

#### State Trace Analysis

The particular implementation of state trace analysis used here is due to Kalish et al. (2016)^2^. In brief, the test examines the rank ordering of the stimuli under both rating types. If the rank orderings are consistent, the two rating outcomes are monotonically related and the state-trace plot is one-dimensional (although not necessarily linear). Otherwise they are not monotonically related, and the state trace plot is two-dimensional. With real-world experimental data, some sampling noise is expected, such that “minor” violations of rank ordering need not necessarily imply the two-dimensional outcome has been obtained. The implementation described by Kalish et al. (2016) takes the one-dimensional result as the null hypothesis, and determines via a non-parametric bootstrap procedure how extreme the observed violation is relative to those found in a bootstrapped population of possible results under the null hypothesis. This quantity is a *p*-value and admits the usual interpretation, with rejection of the null corresponding to a conclusion that the state-trace is two dimensional with a type-I error rate determined by the chosen alpha. No assumptions regarding the data-generating distributions are required. There is a minimum number of data points to avoid degenerate re-sampling in the bootstrap (found in simulation to be *n*≈8), a requirement that is met by this data set.

**Figure 5:**
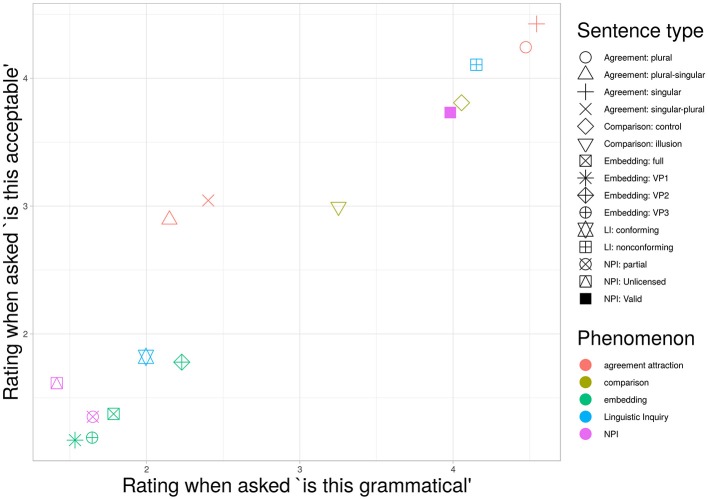
State trace plot. The state-trace analysis used here asks if the rank ordering of two outcome variables is consistent, the visual signature of which is a monotonic relationship when one is plotted against the other. Here, the ratings under the two different instruction conditions do appear monotonically related, although interpreted as such the relationship is not linear.

The eponymous state-trace plot is given in Figure 5. On inspection, the points appear to lie on a one-dimensional S-shaped curve. The *p*-value associated with this configuration is *p* = 0.18, failing to reject the one-dimensional null hypothesis.

### Discussion

Given this pattern of responses, what can be concluded about the impact of the instruction differences? How does this contribute to a more complete description of rating task behavior, and in particular the potential disconnect between what linguists want to know and how participants do the task?

First, the results suggest that people are quite sensitive to the wording of rating task instructions. Ratings for the same sentences differed across the two question blocks as a result of instruction wording. Not only were responses in the “grammatical” question block more extreme (which might also be expected from a simple demand effect), people's ratings differentiated between at least two of the probe phenomena in the opposing directions. Errors of agreement attraction were rated more leniently under acceptability instructions and center-embedding sentences were rated more leniently under grammaticality instructions. The impact of the instructions cannot be a simple scalar shift in lenience or a change in the volatility of ratings. However, despite producing differences with opposite signs, the ratings across the two instruction conditions remained consistent with a one-dimensional state trace. The relevance of the response to the instruction manipulation to the theoretical distinction between grammaticality and acceptability lies not in any direct mapping between the two but rather by requiring accounts of the rating process to accommodate both the differential impact of the instruction manipulation on different sentence types while maintaining a single underlying dimension on which they vary.

The signal detection model of rating behavior proposed by Bader and Häussler (2010) is one such account. Under this description of the rating task, the impact of the instruction manipulation could be described as a reduction of noise and an increase in caution when rating under “grammaticality” instructions relative to “acceptability” instructions. The slight increase in response times observed for the “grammaticality” questions is also consistent with this interpretation. The viability of the signal detection model undermines arguments that grammatical illusion phenomena demonstrate the need to appeal to multiple qualitatively different factors in lay ratings of sentences. It is not the case that dissociations between grammatical status and acceptability rating are only produced by experts working under a technical definition of grammaticality: naive participants in this study also sometimes produce such dissociations for lay interpretations of grammaticality. It remains possible given these data that the two are related, and that the limiting tendency of an ideal acceptability judgment under noiseless conditions and high caution may potentially reproduce the expert pattern without necessarily invoking distinct latent components of ratings. Further, “expert-like” and “acceptability-like” patterns may be apparent to the same people at the same time, as they were to the participants in this study, if the underlying goodness quality is interrogated in different ways, as occasioned in this example by simply changing the wording of the question.

Arguments from the subjective experience of dissociating judgments in grammatical illusion phenomena are not the only evidence for a separation between latent components of acceptability ratings. Arguments highlighting systematic deficits in the performance of language models trained to predict acceptability judgement without recourse to an explicitly separate syntactic information (Dyer et al., 2016; Sprouse et al., 2018; Warstadt and Bowman, 2019; Warstadt et al., 2019) suggest endorsing the “component” interpretation. However, whether these deficits are inherent to all such approaches or simply reflect the peculiarities of current state of the art is an open question. The effect of the instruction manipulation presented here on ratings suggests that an alternative argument from grammatical illusion phenomena is unsound: it is possible that subjective dissociations between canonical status and acceptability rating can be accommodated by appealing to *unbiased* noise and caution only. We do not claim that participants were producing judgments of grammaticality in the technical sense when asked to “rate for grammaticality,” but we observe that whatever the change in the decision making process was, it moved rating judgments toward the outcomes that would be expected from expert analysis and did so by a mechanism consistent with increased deliberation only.

The results are subject to a number of caveats. Most importantly, the one-dimensional state trace result is subject to the usual cautions against interpreting a failure to reject as evidence for the null: it may be that the fifteen sentence types represented in this study are simply not diagnostic. The phenomena used here were chosen to maximize the chance of finding a dissociation between structural and other features, but it is definitely possible that a broader survey of the stimulus space would identify a dissociation where these sentences did not.

The results only apply to the particular population sampled. Because the outcomes rely heavily on the culturally-bound interpretation of the instructions, this study is tightly constrained by the limitations of WEIRD participant pools (Henrich et al., 2010), such as Mechanical Turk workers (Levay et al., 2016). The results of the instruction manipulation may depend on education level, age cohort, or handedness, and the analyses presented here provide no mechanism for identifying systematic effects due to such factors or mitigating them if found. In particular, any differences in ratings due to education up to and including linguistic-specific expertise would be highly desirable, but these data do not support such an analysis.

It's not clear which elements of the instruction manipulation were responsible for producing the differences in ratings. Candidate elements include the description of the judgment types, the colorless green ideas example, the attention check quiz associated with the instructions, and the labeling of the response options. In particular, the decision to express “grammaticality judgments” as a rating of confidence in the presence or absence of errors may have encouraged a different pattern of results to that which would have been obtained under some alternative set of response options. We considered matching the numerical range of the two rating scales to be the conservative choice when testing for differences between them.

One motivation for this work was to quantify the extent to which detailed instructions can help control variability in the motivation behind ratings identified by Schütze (in press). Although the instructions were quite brief and participants on the Mechanical Turk platform are often highly motivated to finish studies quickly, we find statistically significant differences in ratings due to the instruction manipulation. Although these data argue against appealing to people's ability to isolate any specific component of overall acceptability, they also show that rating tasks drawing attention to structural components of acceptability specifically can produce qualitative differences in ratings that may be meaningful on the scale of typical effect sizes in linguistics. The main contribution of this study is to point out that there is no contradiction between these two things, they can both be true at the same time.

## Data Availability Statement

The datasets generated for this study are available on request to the corresponding author.

## Ethics Statement

The studies involving human participants were reviewed and approved by University of Michigan Health Sciences and Behavioral Sciences review board, irbhsbsumich.edu. The patients/participants provided their written informed consent to participate in this study.

## Author Contributions

SL designed the study, collected the data, and drafted the initial write-up under the supervision of RL, who contributed to all stages of this process. RS and JD supplied the code and documentation used in the analysis, reviewed the analysis, and contributed significantly to editing the initial draft for clarity and correctness.

### Conflict of Interest

The authors declare that the research was conducted in the absence of any commercial or financial relationships that could be construed as a potential conflict of interest.

## Footnotes

^1^While the colorless green ideas sentence was originally presented as a dissociation between surface probability and structural soundness, modern approaches to language modeling generally agree that the grammatical permutation of these words is indeed more probable than the alternatives (Abney, 1996; Pereira, 2000; Manning, 2002). Accepting this caveat, the sentence remains a striking example of a dissociation between structural soundness and plausibility, and we considered it a good vehicle for communicating the basic idea of isolating judgments of grammatical structure to participants.

^2^Using the implementation https://github.com/michaelkalish/STA (accessed February 25, 2019).
