# Supplementary material for: In Search of the Factors Behind Naive Sentence Judgments: A State Trace Analysis of Grammaticality and Acceptability Ratings
Source: Front Psychol. 2019 Dec 20;10:2886. doi: 10.3389/fpsyg.2019.02886 (PMC6934035; doi:10.3389/fpsyg.2019.02886)
Supplement: Supplementary file 1 [file Data_Sheet_1.pdf]

Appendix A
Full instructions

There are probably lots of things that contribute to how ’good’ a sentence sounds. These factors include how easy it is to process, whether it makes sense, and whether or not it’s grammatical.

In this study, we’re looking at grammar specifically and how that relates to overall acceptability.

There will be two blocks of questions. One will ask you to rate how acceptable a sentence is, and the other will ask you to indicate if a sentence is grammatical or not.

A sentence is **grammatical** if it ‘follows the rules’ for constructing an English sentence. A sentence doesn’t have to be a good sentence, or even make any sense at all to be grammatical. The most famous example of this is the sentence ‘colorless green ideas sleep furiously’. Even though this sentence doesn’t make sense, it ‘follows the rules’ for constructing an English sentence. English speakers mostly agree that ’colorless green ideas sleep furiously’ is grammatical while ’green furiously ideas colorless sleep’ is not.

In the grammar block, please use the buttons on the screen to say how confident you are that the sentence is grammatical or ungrammatical. Please label all sentences with a grammatical error as ungrammatical, even if the error is small, and label all sentences with no errors as grammatical, even if they are badly written or unclear.

In contrast, **acceptability** is a broader concept that is more about how natural a sentence sounds.

Among all grammatical sentences, some will be highly acceptable and ‘sound good’ while others will be not very acceptable and ‘sound bad’, even though they’re all grammatical. Similarly, although ungrammatical sentences tend to ‘sound bad’, some are worse than others.

In the acceptability block, please rate each sentence on the acceptability scale provided. Sentences that sound natural should get higher ratings, sentences that sound bad should get lower ratings.

Appendix B
Stimuli

The ancient manuscript that the grad student who the new card catalog had confused a great deal was studying in the library was missing a page.
The ancient manuscript that the grad student who the new card catalog was studying in the library was missing a page.
The ancient manuscript that the grad student who the new card catalog had confused a great deal was missing a page.
The ancient manuscript that the grad student who the new card catalog had confused a great deal was studying in the library.
The lullaby that the famous country singer who the record label had signed to a big contract was singing yesterday was written seventy years ago.
The lullaby that the famous country singer who the record label was singing yesterday was written seventy years ago.
The lullaby that the famous country singer who the record label had signed to a big contract was written seventy years ago.
The lullaby that the famous country singer who the record label had signed to a big contract was singing yesterday.
The game that the child who the lawnmower had startled in the yard was playing in the morning lasted for hours.
The game that the child who the lawnmower was playing in the morning lasted for hours.
The game that the child who the lawnmower had startled in the yard lasted for hours.
The game that the child who the lawnmower had startled in the yard was playing in the morning.
The crime that the gangster who the story had profiled had planned for weeks was quickly solved.
The crime that the gangster who the story had planned for weeks was quickly solved.
The crime that the gangster who the story had profiled was quickly solved.
The crime that the gangster who the story had profiled had planned for weeks.
The picture that the artist who the school had expelled for cheating was hurriedly copying was printed in a magazine.
The picture that the artist who the school was hurriedly copying was printed in a magazine.
The picture that the artist who the school had expelled for cheating was printed in a magazine.
The picture that the artist who the school had expelled for cheating was hurriedly copying.
The trophy that the athlete who the restaurant had hired as a spokesman had won at the track meet was stolen later.
The trophy that the athlete who the restaurant had won at the track meet was stolen later.
The trophy that the athlete who the restaurant had hired as a spokesman was stolen later.
The trophy that the athlete who the restaurant had hired as a spokesman had won at the track meet.
The apartment that the maid who the service had sent over was cleaning every week was well decorated.
The apartment that the maid who the service was cleaning every week was well decorated.
The apartment that the maid who the service had sent over was well decorated.
The apartment that the maid who the service had sent over was cleaning every week.
The shirt that the seamstress who the immigration officer had investigated last week was carefully mending needed to be dry cleaned.
The shirt that the seamstress who the immigration officer was carefully mending needed to be dry cleaned.
The shirt that the seamstress who the immigration officer had investigated last week needed to be dry cleaned.
The shirt that the seamstress who the immigration officer had investigated last week was carefully mending.
The lecture that the professor who the newspaper story had just profiled in detail was teaching poorly was not well attended.
The lecture that the professor who the newspaper story was teaching poorly was not well attended.
The lecture that the professor who the newspaper story had just profiled in detail was not well attended.
The lecture that the professor who the newspaper story had just profiled in detail was teaching poorly.

The novel that the horror author who the publishing company had recently fired had typed quickly was banned by the local library.
The novel that the horror author who the publishing company had typed quickly was banned by the local library.
The novel that the horror author who the publishing company had recently fired was banned by the local library.
The novel that the horror author who the publishing company had recently fired had typed quickly.
The prayer that the monk who the religious fanatic had persecuted relentlessly was chanting every day was echoing in the empty church.
The prayer that the monk who the religious fanatic was chanting every day was echoing in the empty church.
The prayer that the monk who the religious fanatic had persecuted relentlessly was echoing in the empty church.
The prayer that the monk who the religious fanatic had persecuted relentlessly was chanting every day.
The monologue that the actor who the movie industry had snubbed repeatedly was performing last month was extremely well written.
The monologue that the actor who the movie industry was performing last month was extremely well written.
The monologue that the actor who the movie industry had snubbed repeatedly was extremely well written.
The monologue that the actor who the movie industry had snubbed repeatedly was performing last month.
The carpenter who the craftsman that the peasant carried hurt supervised the apprentice.
The carpenter who the pillar that the peasant carried hurt supervised the apprentice.
The carpenter who the craftsman that the peasant carried supervised the apprentice.
The carpenter who the pillar that the peasant carried supervised the apprentice.
The mother who the daughter that the sister found frightened greeted the grandmother.
The mother who the gun that the sister found frightened greeted the grandmother.
The mother who the daughter that the sister found greeted the grandmother.
The mother who the gun that the sister found greeted the grandmother.
The worker who the tenant that the foreman looked for injured questioned the shepherd.
The worker who the bucket that the foreman looked for injured questioned the shepherd.
The worker who the tenant that the foreman looked for questioned the shepherd.
The worker who the bucket that the foreman looked for questioned the shepherd.
The trader who the businessman that the professor hired confused annoyed the investor.
The trader who the computer that the professor hired confused annoyed the investor.
The trader who the businessman that the professor hired annoyed the investor.
The trader who the computer that the professor hired annoyed the investor.
The painter who the musician that the father missed sheltered cooked for the artist.
The painter who the hut that the father missed sheltered cooked for the artist.
The painter who the musician that the father missed cooked for the artist.
The painter who the hut that the father missed cooked for the artist.
The saxophonist who the trumpeter that the conductor brought along distracted thanked the violinist.
The saxophonist who the baton that the conductor brought along distracted thanked the violinist.
The saxophonist who the trumpeter that the conductor brought along thanked the violinist.
The saxophonist who the baton that the conductor brought along thanked the violinist.
The pharmacist who the optician that the stranger saw troubled questioned the customer.
The pharmacist who the button that the stranger saw troubled questioned the customer.
The pharmacist who the optician that the stranger saw questioned the customer.
The pharmacist who the button that the stranger saw questioned the customer.
The cleaner who the janitor that the doctor recognised hurt surprised the patient.
The cleaner who the ball that the doctor recognised hurt surprised the patient.
The cleaner who the janitor that the doctor recognised surprised the patient.
The cleaner who the ball that the doctor recognised surprised the patient.
The dancer who the singer that the bystander admired hurt tipped the doorman.
The dancer who the shoe that the bystander admired hurt tipped the doorman.

The dancer who the singer that the bystander admired tipped the doorman.
The dancer who the shoe that the bystander admired tipped the doorman.
The artist who the sportsman that the guard shouted at annoyed instructed the newscaster.
The artist who the computer that the guard shouted at annoyed instructed the newscaster.
The artist who the sportsman that the guard shouted at instructed the newscaster.
The artist who the computer that the guard shouted at instructed the newscaster.
The clerk who the bureaucrat that the visitor forgotten about helped annoyed the neighbour.
The clerk who the walking stick that the visitor forgotten about helped annoyed the neighbour.
The clerk who the bureaucrat that the visitor forgotten about annoyed the neighbour.
The clerk who the walking stick that the visitor forgotten about annoyed the neighbour.
The son who the father that the teacher saw disturbed visited the grandfather.
The son who the loudspeaker that the teacher saw disturbed visited the grandfather.
The son who the father that the teacher saw visited the grandfather.
The son who the loudspeaker that the teacher saw visited the grandfather.
The conductor who the choirmaster that the worker ignored hit berated the musician.
The conductor who the sponge that the worker ignored hit berated the musician.
The conductor who the choirmaster that the worker ignored berated the musician.
The conductor who the sponge that the worker ignored berated the musician.
The defence who the prosecutor that the spy looked at surprised convinced the judge.
The defence who the knife that the spy looked at surprised convinced the judge.
The defence who the prosecutor that the spy looked at convinced the judge.
The defence who the knife that the spy looked at convinced the judge.
The cousin who the brother that the peasant described pleased hated the uncle.
The cousin who the diamond that the peasant described pleased hated the uncle.
The cousin who the brother that the peasant described hated the uncle.
The cousin who the diamond that the peasant described hated the uncle.
The painter who the musician that the friend liked disturbed admired the poet.
The painter who the film that the friend liked disturbed admired the poet.
The painter who the musician that the friend liked admired the poet.
The painter who the film that the friend liked admired the poet.
The slogan on the poster is offensive to vegetarians.
The slogan on the posters are offensive to vegetarians.
The slogans on the poster is offensive to vegetarians.
The slogans on the posters are offensive to vegetarians.
The label on the bottle is quite hard to read.
The label on the bottles are quite hard to read.
The labels on the bottle is quite hard to read.
The labels on the bottles are quite hard to read.
The name on the enormous highway billboard belongs to a local real-estate agent.
The name on the enormous highway billboards belong to a local real-estate agent.
The names on the enormous highway billboards belongs to a local real-estate agent.
The names on the enormous highway billboards belong to a local real-estate agent.
The picture on the postcard is of a Dutch farm.
The picture on the postcards are of a Dutch farm.
The pictures on the postcard is of a Dutch farm.
The pictures on the postcards are of a Dutch farm.
The problem in the wealthy suburban school has been brewing for years.
The problem in the wealthy suburban schools have been brewing for years.
The problems in the wealthy suburban school has been brewing for years.
The problems in the wealthy suburban schools have been brewing for years.
The defect in the car is not obvious except to experts.
The defect in the cars are not obvious except to experts.
The defects in the car is not obvious except to experts.

The defects in the cars are not obvious except to experts.
The mistake in the program is small but important.
The mistake in the programs are small but important.
The mistakes in the program is small but important.
The mistakes in the programs are small but important.
The crime that troubled the detective was not what you might expect.
The crime that troubled the detectives were not what you might expect.
The crimes that troubled the detective was not what you might expect.
The crimes that troubled the detectives were not what what you might expect.
The memo from the accountant flutters to the floor unnoticed.
The memo from the accountants flutter to the floor unnoticed.
The memos from the accountant flutters to the floor unnoticed.
The memos from the accountants flutter to the floor unnoticed.
The letter from the real estate company's lawyer is short and polite.
The letter from the real estate company's lawyers are short and polite.
The letters from the real estate company's lawyers is short and polite.
The letters from the real estate company's lawyers are short and polite.
The warning from the expert falls on deaf ears.
The warning from the experts fall on deaf ears.
The warnings from the expert falls on deaf ears.
The warnings from the experts fall on deaf ears.
The check from the indicted Wall Street stockbroker bounces, as you expected.
The check from the indicted Wall Street stockbrokers bounce, as you expected.
The checks from the indicted Wall Street stockbroker bounces, as you expected.
The checks from the indicted Wall Street stockbrokers bounce, as you expected.
The key to the cabinet is on the table.
The key to the cabinets are on the table.
The keys to the cabinet is on the table.
The keys to the cabinet are on the table.
The door to the President's private office is rarely open.
The door to the President's private offices are rarely open.
The doors to the President's private office is rarely open.
The doors to the President's private offices are rarely open.
The bridge to the island is very crowded.
The bridge to the islands are very crowded.
The bridges to the island is very crowded.
The bridges to the islands are very crowded.
The entrance to the world-famous biology laboratory is visible from the street.
The entrance to the world-famous biology laboratories are visible from the street.
The entrances to the world-famous biology laboratory is visible from the street.
The entrances to the world-famous biology laboratories are visible from the street.
The tile used to cover the floor is from Morocco.
The tile used to cover the floors are from Morocco.
The tiles used to cover the floors is from Morocco.
The tiles used to cover the floors are from Morocco.
The guard employed for the arms-reduction treaty signing ceremony was from a neutral third country.
The guard employed for the arms-reduction treaty signing ceremonies were from a neutral third country.
The guards employed for the arms-reduction treaty signing ceremony was from a neutral third country.
The guards employed for the arms-reduction treaty signing ceremonies were from a neutral third country.
The actor hired to do the commercial is boring and uninspired.
The actor hired to do the commercials are boring and uninspired.
The actors hired to do the commercial is boring and uninspired.
The actors hired to do the commercials are boring and uninspired.

The computer installed in the missile is less powerful than your phone.
The computer installed in the missiles are less powerful than your phone.
The computers installed in the missile is less powerful than your phone.
The computers installed in the missile are less powerful than your phone.
The mechanic who repaired the limousine's rear tire is in a feud with my cousin's friend's family.
The mechanic who repaired the limousine's rear tires are in a feud with my cousin's friend's family.
The mechanics who repaired the limousine's rear tire is in a feud with my cousin's friend's family.
The mechanics who repaired the limousine's rear tires are in a feud with my cousin's friend's family.
The detective who solved the murder is at the door.
The detective who solved the murders are at the door.
The detectives who solved the murders is at the door.
The detectives who solved the murders are at the door.
The professor who criticized the new dean accepts the new era grudgingly.
The professor who criticized the new deans accept the new era grudgingly.
The professors who criticized the new dean accepts the new era grudgingly.
The professors who criticized the new deans accept the new era grudgingly.
The receptionist who greeted the distinguished visitor was fluent in Spanish..
The receptionist who greeted the distinguished visitors were fluent in Spanish.
The receptionists who greeted the distinguished visitor was fluent in Spanish.
The receptionists who greeted the distinguished visitors were fluent in Spanish.
The boy that liked the colorful garter snake hides motionless behind a tree.
The boy that liked the colorful garter snakes hide motionless behind a tree.
The boys that liked the colorful garter snake hides motionless behind a tree.
The boys that liked the colorful garter snakes hide motionless behind a tree.
The astronomer that discovered the most distant galaxy is now famous.
The astronomer that discovered the most distant galaxies are now famous.
The astronomers that discovered the most distant galaxy is now famous.
The astronomers that discovered the most distant galaxies are now famous.
The table that the student painted looks nice.
The table that the students painted look nice.
The tables that the student painted looks nice.
The tables that the students painted look nice.
The girl that the science teacher questioned was nervous.
The girl that the science teachers questioned were nervous.
The girls that the science teacher questioned was nervous.
The girls that the science teachers questioned were nervous.
The soldier that the battalion's senior officer accused was innocent.
The soldier that the battalion's senior officers accused were innocent.
The soldiers that the battalion's senior officer accused was innocent.
The soldiers that the battalion's senior officers accused were innocent.
The policy that the governor recommended was hugely unpopular.
The policy that the governors recommended were hugely unpopular.
The polices that the governor recommended was hugely unpopular.
The policies that the governors recommended were hugely unpopular.
The dog that chased the truck is very muddy.
The dog that chased the trucks are very muddy.
The dogs that chased the truck is very muddy.
The dogs that chased the trucks are very muddy.
No restaurants that local newspapers have recommended in their dining reviews have ever gone out of business.
The restaurants the no local newspapers have recommended in their dining reviews have ever gone out of business.

Most restaurants the local newspapers have recommended in their dining reviews have ever gone out of business.
No pirate with a beard has ever beaten me at cards.
A pirate with no beard has ever beaten me at cards.
Every pirate with no beard has ever beaten me at cards.
No cricket player from Australia has ever run faster than Ponting.
Many cricket players who are not from Australia have ever run faster than Ponting.
Most cricket players from Australia have ever run faster than Ponting.
No performance involving this style of mask has ever been traditional on Palau.
A performance not involving this style of mask has ever been traditional on Palau.
Each performance involving this style of mask has ever been traditional on Palau.
Few gamblers with a system have ever finished ahead.
A gambler without a system has ever finished ahead.
Most gamblers with a system have ever finished ahead.
Not many skyscrapers from the 80's ever brought their designers fame.
Many skyscrapers not from the 80's ever brought their designers fame.
The skyscrapers from the 80's ever brought their designers fame.
The main advantage of microchipping your pet is that few pets with microchips are ever lost for long.
The main advantage of microchipping your pet is that pets without microchips are ever lost for long.
The main advantage of microchipping your pet is that pets with microchips are ever lost for long.
No superhero with a cape has ever missed a chance to flaunt it.
A superhero without a cape has ever missed a chance to flaunt it.
Every superhero with a cape has ever missed a chance to flaunt it.
No swim instructors with experience would ever recommend underwater distance training.
Some swim instructors without experience would ever recommend underwater distance training.
Some swim instructors with experience would ever recommend underwater distance training.
Only a mad captain with a plan ever gives that order.
A mad captain without a plan ever gives that order.
The mad captain with a plan ever gives that order.
Not many musicians who could play sitar ever recorded their most popular works.
Many musicians who never played sitar ever recorded their most popular works.
Many musicians who could play sitar ever recorded their most popular works.
None of the garden forks with wooden handles ever have more than four tines.
All of the garden forks without wooden handles ever have more than four tines.
All of the garden forks with wooden handles ever have more than four tines.
None of the teapots genuinely from the Victorian era ever have a maker's mark underneath.
The teapots not genuinely from the Victorian era ever have a maker's mark underneath.
The teapots genuinely from the Victorian era ever have a maker's mark underneath.
No calligraphy except that by Mi Fu ever bears that particular seal.
Most calligraphy not by Mi Fu ever bears that particular seal.
Most calligraphy by Mi Fu ever bears that particular seal.
Nobody who believed ever said so much as a word about it.
Someone who didn't believe ever said so much as a word about it.
Everyone who believed ever said so much as a word about it.
Nobody other than the vegetarian ate any spinach.
A man other than the vegetarian ate any spinach.
A man ate any spinach.
No authors that the critics recommended have received any acknowledgement for a best selling novel.
The authors that no critics recommended have received any acknowledgement for a best selling novel.
The authors that the critics recommended have received any acknowledgement for a best selling novel.
No party that was organized by the journalists had any problems with catering.
The party that was not organized by the journalists had any problems with catering.
The party that was organized by the journalists had any problems with catering.

No singer that the venue hosted had any problem selling tickets.
The singer that the venue couldn’t host had any problem selling tickets.
The singer that the venue hosted had any problem selling tickets.
No cake that the children found had any chance of surviving the week.
The cake that the children didn’t find had any chance of surviving the week.
The cake that the children found had any chance of surviving the week.
No camel breeders from Bikaner have received any prizes that season.
The camel breeders not from Bikaner have received any prizes that season.
The camel breeders from Bikaner have received any prizes that season.
No voice actors who do major cartoons get any fans approaching them in the street.
Voice actors who do not do major cartoons get any fans approaching them in the street.
Voice actors who do major cartoons get any fans approaching them in the street.
Few islands that have ground birds have any rats.
Islands that don’t have ground birds have any rats.
Every island that has ground birds has any rats.
Last summer more famous bands had a big stadium show than lesser-known bands did.
Last summer more famous bands had a big stadium show than the lesser-known band did.
In English class more girls wrote a charming haiku than boys did.
In English class more girls wrote a charming haiku than I did.
This year more football fans bought a home game ticket than hockey fans did.
This year more football fans bought a home game ticket than the hockey fan did.
At the new cafe more women tried a new green tea drink than reluctant men did.
At the new cafe more women tried a new green tea drink than the reluctant man did.
At Peter’s party more toddlers ate a tasty strawberry cupcake than high schoolers did.
At Peter’s party more toddlers ate a tasty strawberry cupcake than the high schooler did.
Before exams ended more undergrads handed in a final paper than grad students did.
Before exams ended more undergrads handed in a final paper than the grad student did.
That year more visiting Americans saw an exciting bullfight than young Spaniards did.
That year more visiting Americans saw an exciting bullfight than the young Spaniard did.
Yesterday more seniors skipped an extremely boring class than juniors did last month.
Yesterday more seniors skipped an extremely boring class than Jonathan did last month.
By 5 o’clock more busy telemarketers had taken an easy call than lazy telemarketers did.
By 5 o’clock more busy telemarketers had taken an easy call than the lazy telemarketer did.
At the bridal shower more family members drank a tall glass of champagne than friends did.
At the bridal shower more family members drank a tall glass of champagne than Fiona did.
That week more female students handed in a short term paper than male students did.
That week more female students handed in a short term paper than the male student did.
Last year more professional writers published an acclaimed novel than amateur authors did.
Last year more professional writers published an acclaimed novel than the amateur author did.
This week more hard-working students continued writing their thesis than lazy students did.
This week more hard-working students continued writing their thesis than the lazy student did.
In September more New Yorkers attended law school than Canadians did all of last year.
In September more New Yorkers attended law school than Candace did all of last year.
During that war more middle-aged Alabamans were exposed to combat than young Alaskans were.
During that war more middle-aged Alabamans were exposed to combat than the young Alaskan was.
That week more German architects continued working on a big project than French architects did.
That week more German architects continued working on a big project than the French architect did.
Last month more junior employees prolonged their vacation than senior employees did.
Last month more junior employees prolonged their vacation than the senior employee did.
Last week more History majors continued writing their literature essay than English majors did.
Last week more History majors continued writing their literature essay than the English major did.
On Monday more librarians organized an enormous filing task than secretaries did on Tuesday.
On Monday more librarians organized an enormous filing task than the secretary did on Tuesday.

Last year more young people were reading War
Peace than old men were.
Last year more young people were reading War
Peace than the old man was.
Over the weekend more boys worked on the assignment than girls did.
Over the weekend more boys worked on the assignment than Jenna did.
In cooking class more international students were baking butter scones than American students were.
In cooking class more international students were baking butter scones than the American student was.
Last month more lawyers were writing their big report than company executives were.
Last month more lawyers were writing their big report than the company executive was.
At the party more seniors were talking with the professors than juniors were.
At the party more seniors were talking with the professors than the junior was.
There has been a man considered sick.
There has been a woman considered desperate.
There has been a man considered violent.
There has been a dog considered noisy.
We proved Susan to the authorities to be the thief.
We alleged Carrie to the police to be guilty.
We alleged Ellen to the judge to be a liar.
Joan bought a last year house next to the house that Erin did.
John wants for everyone to have fun that you do.
Dana hopes for everyone to succeed that you do.
Melanie prefers for everyone to get a raise that you do.
Kyle expects for everyone to be honest that you do.
Sophia is anxious for everyone to arrive that you are.
Ben is hopeful for everyone to attend that you are.
Valerie is excited for everyone to graduate that you are.
What do you complain that the neighbor turns on at night?
Sarah gave more children than Susan did candy.
Kimberly wanted to give the charity something warm to wear, and give the charity a bundle of jackets she did.
They knew and we saw that Mark would skip work.
Mary believed that Peter finished school and Bill that Peter got a job.
Sarah believed that Daniel grew a few inches and Richard that Daniel gained some weight.
Raymond believed that Amy practiced her music and Danielle that Amy won a competition.
That Mike pushed the little girl, Bianca didn’t believe.
That Leonard drank the whole bottle, Michael didn’t believe.
That Addison bit the boy, Jena didn’t believe.
I believed a friend of Andy satisfied.
I expect that everyone will visit Mary that you do.
I anticipate that everyone will see John that you do.
We expect that everybody will hug Kimberly that you do.
I expect that everyone will praise Ricky that you do.
I anticipate that everybody will visit Jack that you do.
They anticipate that everybody will contact Fred that you do.
He anticipates that everyone will meet Susie that you do.
I anticipate that everybody will watch Melissa that you do.
There is likely to spread a disease around the world.
There is likely to depart a train at midnight.
There is likely to live a snake in the garden.
There is likely to run a river down the mountain.
Down which street drove two cars?
Larry cooked her husband the meal.

He envied me my success after the promotion.
That movie impossible to watch without enjoying.
The club was entered wearing no shirt.
The bed was slept in wearing no clothes.
The summit was reached sweating profusely.
The papers were graded while being bored.
The car was driven while being tired.
The question was answered feeling nervous.
We were sure that the teaching assistant liked to meet before lecture.
There had prevailed in the trial all of the defendants we were rooting for.
There had lived across the street all of the kids who were vandalizing our yard.
Who did they send a parent of to an unpleasant meeting?
Who did the coach trade an enemy of to another team?
Who did the principal assign children of to a different teacher?
Who did the frat boys introduce a daughter of to their pledges?
Leslie is certain that these clothes Harold will wash.
Judah confirmed that this jersey he can keep.
Mindy was confident that my history notes she could borrow.
Jessie knows that the big solo Rachel will sing.
When this week he started to perform that play, we believed he would be fine.
John believes without a doubt his team will win.
Emma thought for a moment her friend was at the door.
Ashley suggested to Michael he should throw a party.
Jessica saw on the news Michael Jackson died.
Amanda hinted to Jack there will be a pop quiz on Monday.
Mark sailed the Caribbean and Cathy flew to the Mediterranean.
Brent signaled to Samantha to excuse himself.
John put more books on the table than Bill did on the floor.
Greg put more onions in the soup than Lana did in the salad.
The instructor put more solutions on the board than the TA did on the handout.
It seemed at that time Mark had quit.
It seemed at that time Clara had been promoted.
It seemed at that time Adam had been fired.
Natalie pushed the window opened.
To whom did you give what?
To whom did you present what?
Who will you give the keys?
The reward doubled to help catch the thief.
All the postal workers seem to have all taken a break at the same time.
The game was played shoeless.
